# Supplementary material for: A hackable, multi-functional, and modular extrusion 3D printer for soft materials
Source: Sci Rep. 2022 Jul 19;12:12294. doi: 10.1038/s41598-022-16008-6 (PMC9296631; doi:10.1038/s41598-022-16008-6)
Supplement: Supplementary file 3 — Supplementary Information 3. [file 41598_2022_16008_MOESM3_ESM.pdf]

A hackable, multi-functional, and modular extrusion 3D printer for soft materials

Supplementary information

Iek Man Lei[1,2], Yaqi Sheng[1,2], Chon Lok Lei[3,4], Cillian Leow[1] and Yan Yan Shery Huang[1,2]*

1 Department of Engineering, University of Cambridge, Cambridge, United Kingdom
2 The Nanoscience Centre, University of Cambridge, Cambridge, United Kingdom
3 Institute of Translational Medicine, Faculty of Health Sciences, University of Macau, Macau
4 Department of Computer Science, University of Oxford, Oxford, United Kingdom

** Corresponding: yysh2@cam.ac.uk*

I. Printer specification

Supplementary Table 1| Table comparing the system developed in this work with the reported open-source extrusion bioprinters. PF127 = Pluronic F127, PAA = polyacrylic acid, CMC = sodium carboxymethyl cellulose, NaAlg = sodium alginate, PCL = Polycaprolactone, hiPSCs = Human induced pluripotent stem cells, and ECM = extracellular matrix.

Ref	Hardware	Geometry inputs	Auxiliary tools	No. of inks tested	Geometry	Applications demonstrated
Hinton et al (2015)[1]	Piston-based extruder with a commercial 3D printer	CAD	Stage heater	4<br>• *NaAlg*<br>• *Fibrinogen*<br>• *Collagen*<br>• *ECM*	bone, heart, vascular-like structures	Freeform structure
Reid et al (2016)[2]		(bioplotter)	-	*hiPSCs, MCF-12a*	(dispenser)	Cell aggregates
Nava et al (2017)[3]		n/a	-	1<br>• *NaAlg*	Line	Cell-laden line
Polley et al (2017)[4]			-	1<br>• *NaAlg*	Gridline	Cell-laden construct
Bessler, et al. (2019)[5]		CAD	-	1<br>• *NaAlg*	Cylinder and rectangle scaffolds	Cell-laden structure
Kahl, et al. (2019)[6]		CAD	-	2<br>• *NaAlg*<br>• *NaAlg* -gelatin	Lattice, cylinder and pyramid scaffolds	Cell proliferation
Ioannidis, et al. (2020)[7]		CAD	-	1<br>• *NaAlg -gelatin*	Line patterns	Cell proliferation and differentiation
Krige et al (2020)[8]		n/a	UV	2<br>• *Gelma*<br>• *sodium hyaluronate*	Line patterns, rocket-shaped construct	Biofilm electrode
Tashman et al. (2021)[9]		n/a	-	1<br>• *Collagen*	Microfluidic network	Perfusable microfluidic network
Engberg et al. (2021)[10]		CAD	Camera, HEPA filter	2<br>• *collagen*<br>• *lamina*	Rectangular	Cell-laden multimaterial structure
Koch et al (2021)[11]		CAD	Syringe heater	3<br>• *NaAlg -gelatin*<br>• *silicone*<br>• *PCL*	Line, lattices	Hybrid material lattice for osteochondral applications
Cadiou et al (2021)[12]		CAD	-	1<br>• *Epoxy*	Dog bone	Composite structure with improved mechanical performance
Leech et al (2021)[13]		CAD	-	3<br>• *Gelatin*<br>• *Chitosan*<br>• *Marmite*	Line grids	n/a
Sanz-Garcia et al (2020)[14]	Pneumatic extruder with a commercial 3D printer	CAD	Syringe heater and cooler	2<br>• PF127<br>• *Gelatin- NaAlg*	Concentric square, circle, pillars, log-pipe	Cell-laden lattices
Feinberg, et al. (2018) [15–17]	Piston-based extruder with existing build plate	CAD	-	1<br>• *NaAlg*	3D constructs, full size model of the human heart	Patient-specific anatomical models

Ravi et al. (2015)[18]	Piston-based extruders with linear stages	CAD	UV,	2<br>• Toothpaste<br>• Black ink	Rectangular, grid	n/a
Xing et al. (2017)[19]		G-code (CAD)	-	1<br>• PF127-*glycerol*	Log-pipe, ring scaffod	n/a
Fitzsimmon, et al.(2018)[20]		CAD	Syringe heater	5<br>• *Gelatin*<br>• *GelMA*<br>• *Gelatin-hyaluronan*<br>• *Gelatin-NaAlg*<br>• *PF127*	Cell-laden line pattern, 3D constructs (cube, cylinder and star), Constructs with channels	HUVEC culture in channels
Yenilmez, et al. (2019)[21]		Picture, CAD	UV module, coaxial printhead	1<br>• *NaAlg*	Cell-laden grid scaffold	Cell-laden construct
Zhang et al. (2019)	Pneumatic extruder with linear stages	n/a	Heating	*1*<br>• *Silver*	Line pattern	Soft actuation
Lanaro et al (2021)[22]		n/a	Heating	2<br>• *PCL*<br>• *PF127*	Lattice	n/a
Shen, et al. (2021)[23]		CAD	-	1<br>• *NaAlg*	Alginate hydrogel with concentration gradient, a femur model	Cell-laden structures, structure with embedded concentration gradients
Peng, et al. (2021)[24]		CAD	Digital light processing (DLP) projector	• *Liquid crystal elastomer*<br>• *Photocurable urethane*<br>• *Silver*	Line, spiral, ring, buzz patterns, rings	Soft actuation, circuit-embedding architectures, strain sensors
Uzel, et al. (2022)[25]		n/a	Line profilometer	*3*<br>• *PF127*<br>• *Gelatin*<br>• *Photo-polymerisable urethane-based materials*	Line patterns, crest motif	Adaptive conformal patterning
Shim et al. (2012)[26]	Pneumatic and piston-based extruders with linear stages	CAD	Syringe heater	2<br>• *PCL*<br>• *NaAlg*	Letters, 2D bone-shaped structure	Porous osteochondral structure
Lee et al. (2017)[27]		CAD	Syringe heater	3<br>• *PLGA*<br>• *Hyaluronic acid*<br>• *HA-alpha-TCP*	Lattice, cyliner scaffold	Cell-laden hybrid lattice
Byrne et al. (2018)[28]	Pneumatic and FDM extruders with a rotational mandrel and commercial 3D printer	CAD	FDM extruder heater	2<br>• *Silicone*<br>• *Thermoplastic elastomer*	Line grids	Soft actuation
Ozbolat, et al. (2014)[29]	Pneumatic extruders and commercial syringe pump with linear stages	CAD	Coaxial printhead	2<br>• *NaAlg*<br>• *Cell spheroid*	Lattice	Cell proliferation
Spiesz, et al. (2019)[30]	Commercial syringe pump with commercial 3d printer	Coordinates	-	1<br>• *NaAlg*	2D patterns	Spatially-controlled model for E.coli bacteria culture
Ghosheh et al (2016)[31]	Commercial syringe pump with linear stages	CAD	UV, camera	1<br>• *PEGDA*	Line patterns	n/a

Fortunato et al. (2021)[32]	Pneumatic extruder with a robotic arm	CAD	-	1<br>• Pluronic acid	Line pattern	Non-planar printing
**This work**	Piston-based extruders with a robotic arm	***4 geometry input options**<br>• Coordinates<br>• Equation<br>• CAD<br>• Picture	• Stage heater<br>• Syringe heater<br>• UV<br>• Camera	***11**<br>• *Hydrogels (PF127, CMC, NaAlg, gelatin, PAA, PEGDA, methyacrylate hydroxylpropyl cellulose, sodium hyaluronate)*<br>• *Silicone elastomer (SE1700)*<br>• *Bioceramic hydrogels (i.e. NaAlg – hydroxyapatite)*<br>• *3T3 suspension*	***Able to construct complex objects**<br>• Multi-material 3D constructs,<br>• 2D embedded vascular-like channels<br>• 3D intricate objects	***Multiple functions apart from in-air printing**<br>• Multimaterial printing<br>• Embedded printing<br>• Liquid dispensing<br>• Printing with variable speed<br>• Non-planar printing<br>• Pick-and-place

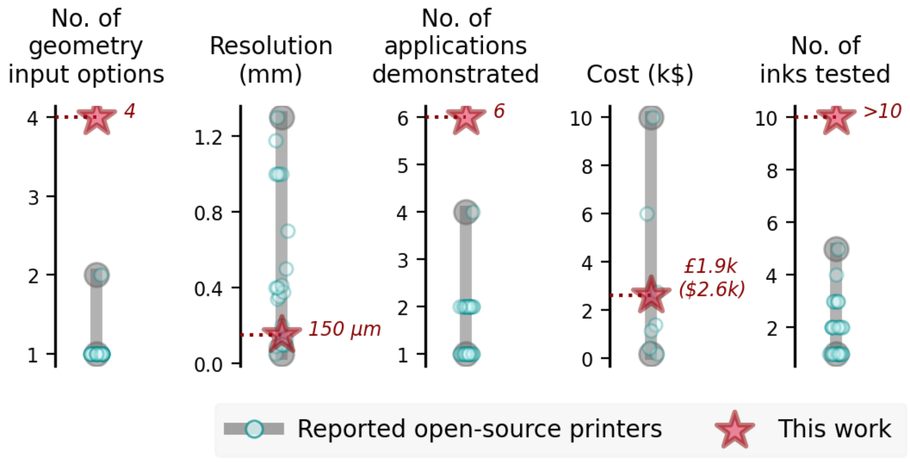

Supplementary Figure 1| A summary comparing work reported in this study, and those reported by existing open-source printer platforms (see above Printer specification for literature source).

Supplementary Table 2| Specifications of the system developed in this study and the four commercial 3D extrusion-based bioprinters commonly used in the bioprinting community. The commercial systems are Allevi 3[33] (denoted as ‡ below), Cellink BioX[34,35] (†) and Envision TEC 3D Bioplotter Starter Series[36] (*) and GeSim BioScaffolder BS3.3[37] (§). It should be noted that, though Printer.HM has a lower mechanical resolution and a narrower heating range than commercial systems, soft material printing typically does not require a high temperature (*i.e.* > 60 °C), and the print resolution of extrusion soft material printing is in general > 100 µm. As Printer.HM is designed for soft material fabrication purpose, the heating range and the mechanical resolution adopted in Printer.HM are considerably adequate.

	**This setup**	**Commercial systems**
Cost	£0.9k (single printhead system) - £1.9k (systems with 4 printheads, syringe and stage heaters, UV and camera)	$10k – 100k [38]
Maximum no. of printhead slots	6 printheads for one Arduino board	• 3 (‡, †)<br>• 2 (*)<br>• 4 (§)
Geometry input	• Coordinates<br>• CAD<br>• Equations<br>• Picture	• CAD (‡, †, *, §)
Mechanical resolution of the motion system	• 200 µm	• 1 µm (‡, †, *)<br>• Information not available (§)
Extruder temperature control	• RT – 60 ºC	• 4 ºC – 160 ºC (‡)<br>• 4 ºC – 65 ºC / 250 ºC (†)<br>• 30 ºC – 250 ºC (*)<br>• 4 ºC – 80 ºC / RT – 190 ºC or 250 ºC (§)
Printbed temperature control	• RT – 60 ºC (tested range)	• RT – 60 ºC (‡)<br>• 4 – 65 ºC (†)<br>• Not available (*, §)
UV power	• 365/405 nm	• Yes (365/405 nm) (‡)<br>• Yes (365/405/450/485/520nm) (†)<br>• No (*)<br>• UV LED (§)
Extrusion method	• Mechanical	• Pneumatic or mechanical (†, §)<br>• Pneumatic (‡, *)
Compatible cartridge size	• 1 ml – 3 ml	• 5 ml (‡)<br>• 3 ml – 10 ml (†)<br>• 3 ml – 30 ml (*)<br>• 10 ml – 30 ml (§)
Weight	• ~11 kg, 45x45x35 cm (excl. enclosure)	• 21.8 kg, 47x40x36 cm (‡)<br>• 18 kg, 48x44x37 cm (†)<br>• 90 kg, 84x62x77 cm (*)<br>• Information not available (§)

II. Bills of materials

Supplementary Table 3| Part list and the breakdown costs of the printing platform. The total cost refers to the associated cost of a platform equipped with 4 printheads, stage and syringe heating systems, a UV module and a camera. * denotes the components used in the electrical circuit.

	Components	Part number	Manufacturer	Cost (£)
**C1**	**uArm Swift Pro Desktop Robotic Arm - Professional Kit**	-	ufactory	**749.98**
	**Frame**			
**C2**	Aluminium rail (20 x 20 x 350 mm) x 4 units	VSLOT2020	Ooznest	10.08
**C3**	Aluminium rail (20 x 40 x 350 mm) x 2 units	VSLOT2040	Ooznest	8.82
**C4**	Breadboard	MB4545/M	Thorlabs	184.52
**C5**	Clamp x 4 units	CL3/M	Thorlabs	14.76
			Subtotal	***203.42***
	**Printhead (Components per printhead)**			
**C6**	Stepper motor, 3.8 V	5350344	RS	31
**C7**	Shaft coupling	PSMR19-5-5-A	Ruland	18
**C8**	Lead screw (107 mm)	DST-LS-6.35x2.54-R-500-ES	Igus	5.15
**C9**	Lead screw nut	DST-JFRM-131315DS6.35X2.54	Igus	17.25
**C10**	Linear rail (100 mm) x 2 units	WSQ-06	Igus	8.62
**C11**	Linear guide pillow block x 2 units	WJ200QM-01-06	Igus	15.74
**C12**	Ball bearing	624ZZ	NSK	3.65
**C13**	Aluminium rail (20 x 40 x 150 mm)	VSLOT2040	Ooznest	1.89
**C14**	Magnet (Height 3 mm diameter 4 mm) x 6 units	M1219-3	Comus	6.12
**C15**	Magnet (Height 2 mm diameter 3 mm) x 6 units	M1219-2	Comus	2.7
**C16**	Stepper motor drive*	A4988	Polulu	7.18
**C17**	Resistors (10 kΩ 0.6 W)*	MF006FF1002KIT	Royal Ohm	1.36
**C18**	Capacitor 100 µF*	EEAGA1A101	Panasonic	0.06
			Subtotal	***118.72***
	**Printhead (Components for whole system)**			
**C19**	Power adapter, 9V, 2A	VEL18US090-UK-JA	XP Power	10.24
**C20**	Arduino mega*	A000067	Arduino	25
**C21**	Breadboard*	TW-E40-1020	Twin Industries	4.13
**C22**	USB A-male to USB B-male cable*	AK-300102-010-S	Digitus	0.42
			Subtotal	***39.79***
	**Heating (Components per module)**			
**C23**	Power adapter, 9V, 2A (syringe heater)<br>Power adapter, 12V, 500mA (stage heater)	VEL18US090-UK-JA/<br>T6116ST	XP Power or<br>Stontronics	10.24/<br>4.02
**C24**	Type K thermocouple	Z2-K-1M	Labfacility	4.64
**C25**	Thermocouple Amplifier (MAX31855K)*	269	Adafruit	12.76
**C26**	MOSFET*	IRLR/U8743PBF	Infineon	1.04
**C27**	Arduino Nano*	A000005	Arduino	14.23
**C28**	Breadboard*	MCO1003	Multicomp	1.10
**C29**	USB A-male to USB mini B-male cable*	ZUV0E3058769	StarTech	1.4
			Subtotal	***~37***
	**Heating (Components for whole system)**			
**C30**	31 AWG Nichrome wire	UMNICWIRE2	Ultimachine	5
**C31**	High temperature tape	051-0002	Antistat	4.27
			Subtotal	***~50***
	**Others**			
**C32**	UV365 LED UV Torch	NSUV365	Nightsearcher	162.5
**C33**	HD Webcam	C922	Logitech	65.1
**C34**	Drop-in Tee Nuts, M5 (~200 units)	VSLOT-H-DT-M5	OOznest	40

	Components	Part number	Manufacturer	Cost (£)
**C35**	MakerLink 90 Degree Hidden Tee Nut x 4 units	VSLOT-H-ML-90H-GS	Openbuilds	4.88
**C36**	Universal L Brackets x 4 units	VSLOT-B-UL-S-C	OOznest	4
**C37**	Jumper cables	MIKROE-511	MikroElektronika	2.41
			Subtotal	***278.89***
			TOTAL	***~1870***

III. Assembly instruction

Prior to the assembly, the custom-designed parts were 3D printed with PLA or ABS using an Ultimaker S3 3D printer. A syringe holder and a stage holder were custom-made with aluminum. All the custom-designed CAD files are listed in **Supplementary Table 4** and are available on Github.

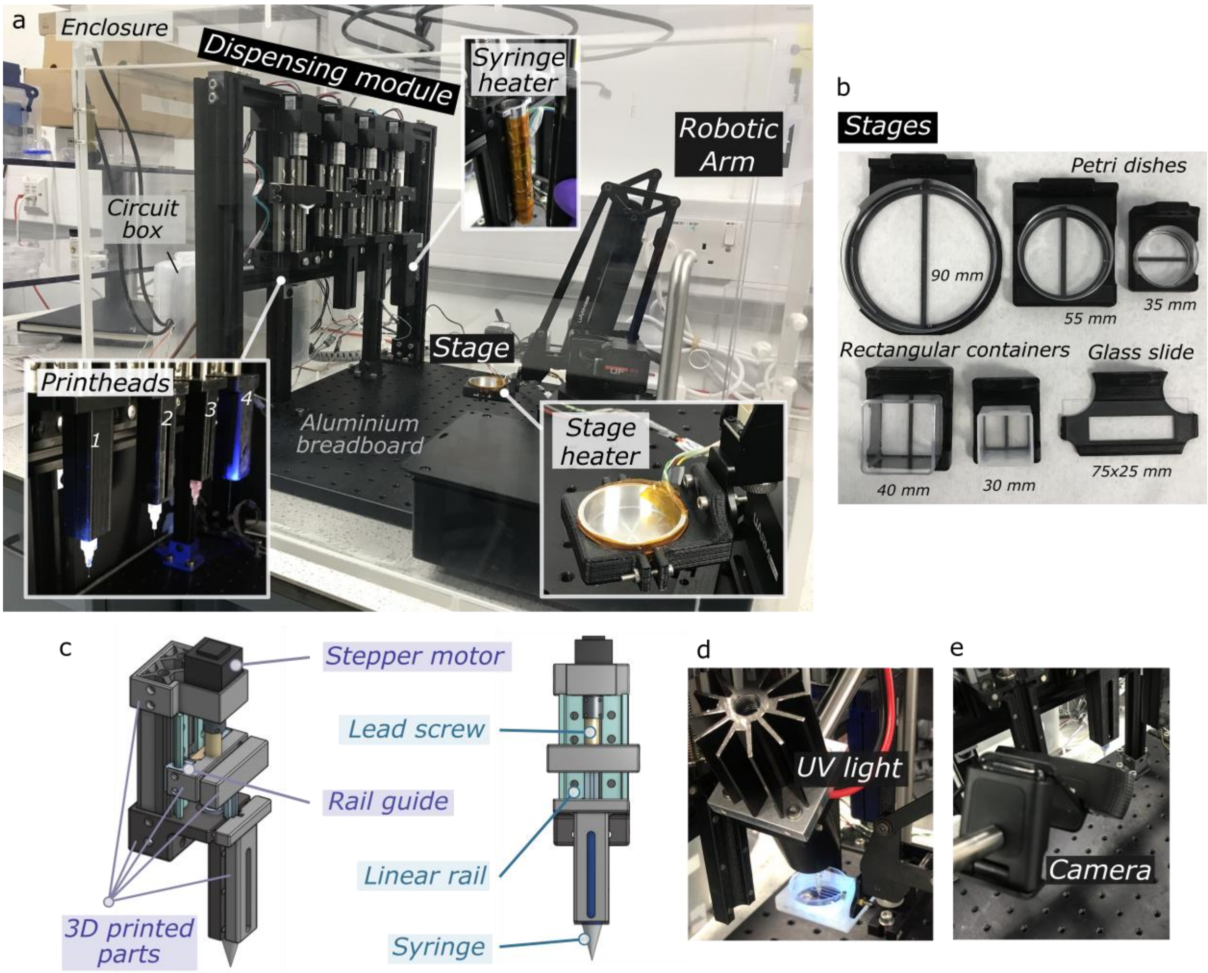

Supplementary Figure 2| a) Overview of the setup. b) 3D printed stages for accommodating different sizes of receiving reservoirs. c) 3D CAD design of the piston-driven extrusion printhead. Add-on d) UV light for photopolymerization and e) camera for *in situ* monitoring.

Supplementary Table 4| Customised accessory list. The printing time is referred to the time required when a 'fast' print setting is used in Ultimaker.

	Components			Materials	Printing time
Frame	**D1**	Rod holder 1 x 2 units	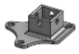	PLA	1 hr
	**D2**	Rod holder 2	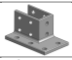	PLA	1 hr 30 min
	**D3**	Rod holder 3	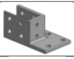	PLA	1 hr 30 min
	**D4**	Adapter plate 1	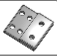	PLA	30 min
	**D5**	Adapter plate 2	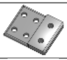	PLA	30 min
	**D6**	Adapter plate 3	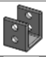	PLA	30 min
	**D7**	Adapter plate 4	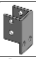	PLA	30 min
	**D8**	Adapter plate 5	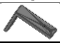	PLA	30 min
Printhead	**D9**	Stepper motor holder	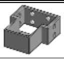	PLA	1 hr
	**D10**	Lead screw adapter_front	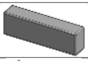	PLA	30 min
	**D11**	Lead screw adapter_back	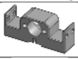	PLA	50 min
	**D12**	Syringe holder_back	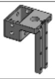	PLA	2 hr
	**D13**	Syringe holder_front	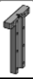	PLA	50 min
Printhead heater	**D14**	Stepper motor holder	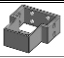	PLA	1 hr
	**D15**	Lead screw adapter_front	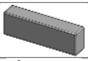	PLA	30 min
	**D16**	Lead screw adapter_back	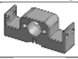	PLA	50 min
	**D17**	Heating syringe holder_back	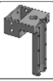	ABS	2 hr 20 min
	**D18**	Heating syringe holder_front	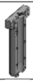	ABS	1 hr 10 min
	**D19**	Syringe heater barrel_AL	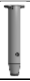	Aluminum	n/a
Stage	**D20**	35 mm petri dish stage	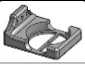	PLA	40 min
	**D21**	55 mm petri dish stage	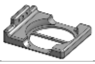	PLA	1 hr 20 min
	**D22**	90 mm petri dish stage	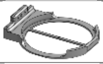	PLA	1 hr 20 min
	**D23**	30 mm rectangular container stage	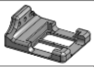	PLA	40 min
	**D24**	38 mm rectangular container stage	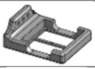	PLA	1 hr
	**D25**	75x25 mm glass slide holder	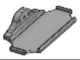	PLA	50 min
Stage heater	**D26**	35 mm petri dish heating stage 1	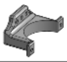	ABS	30 min
	**D27**	35 mm petri dish heating stage 2	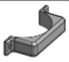	ABS	30 min
	**D28**	35 mm petri dish holder_AL	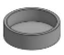	Aluminium	n/a

II.1 Printhead assembly

Supplementary Figure 3 shows the step-by-step assembly instruction of a printhead. **1.** Before assembling the printhead, the lead screw *(Part C8)* was milled to the designed dimension. (see **Supplementary Figure 3**, step 1). **2.** Next, the stepper motor *(Part C6)* was attached to the 3D printed motor holder using M2.5 screws. **3.** The stepper motor holder was then inserted to an aluminium rail *(Part C2)* and locked in place with the drop in tee nuts *(Part C34)* and M5 screws. **4.** A shaft coupling *(Part C7)* was connected to the motor shaft and tightened using the built-in set screw. **5.** Two 10 cm linear rails *(Part C10)* were tightened firmly on the aluminium rail using M5 screws at a position of ~28 mm from the top edge of the aluminium rail. **6.** A linear guide pillow block *(Part C11)* was inserted to each linear rail. **7.** The modified lead screw was combined with a lead screw nut *(Part C9)*, which was then tightened on a 3D printed lead screw nut mount. **8.** The lead screw was tightened to the shaft coupling, and the 3D printed lead screw nut mount was then linked to the linear guide pillow blocks on the linear rail using M4 screws. **9.** The end of the lead screw was connected to a ball bearing *(Part C12)* that was fit to a 3D printed syringe holder. **10.** The 3D printed syringe holder was then tightened on the aluminium rail using M5 screws and the drop in Tee nuts. **11.** The printhead assembly was finished by placing magnets *(Parts C14 & C15)* to the designed holes of the lead screw nut mounts and the syringe holders using super glue.

The heating printhead was assembled similarly using different 3D printed parts of syringe holders *(Parts D17 & D18)* that were custom-made to fit an aluminium barrel *(Part D19)*. The aluminium barrel was wrapped with a Nichrome wire as the heating wire *(Part C30)* and a K-type thermocouple *(Part C24)* as the temperature sensor using a high temperature tape *(Part C31)*.

II.2 Installation of printheads to the frame

The frame of the dispensing module is made of six aluminium rails, and was assembled using hidden tee nuts and L brackets *(Parts C35 & C36)* as linkages according to **Supplementary Figure 3**. The frame was then fastened on the aluminium breadboard by means of the customised 3D printed adapters. In total, 4 printheads were built. They were firmly installed to the frame using the 3D printed adapter plates.

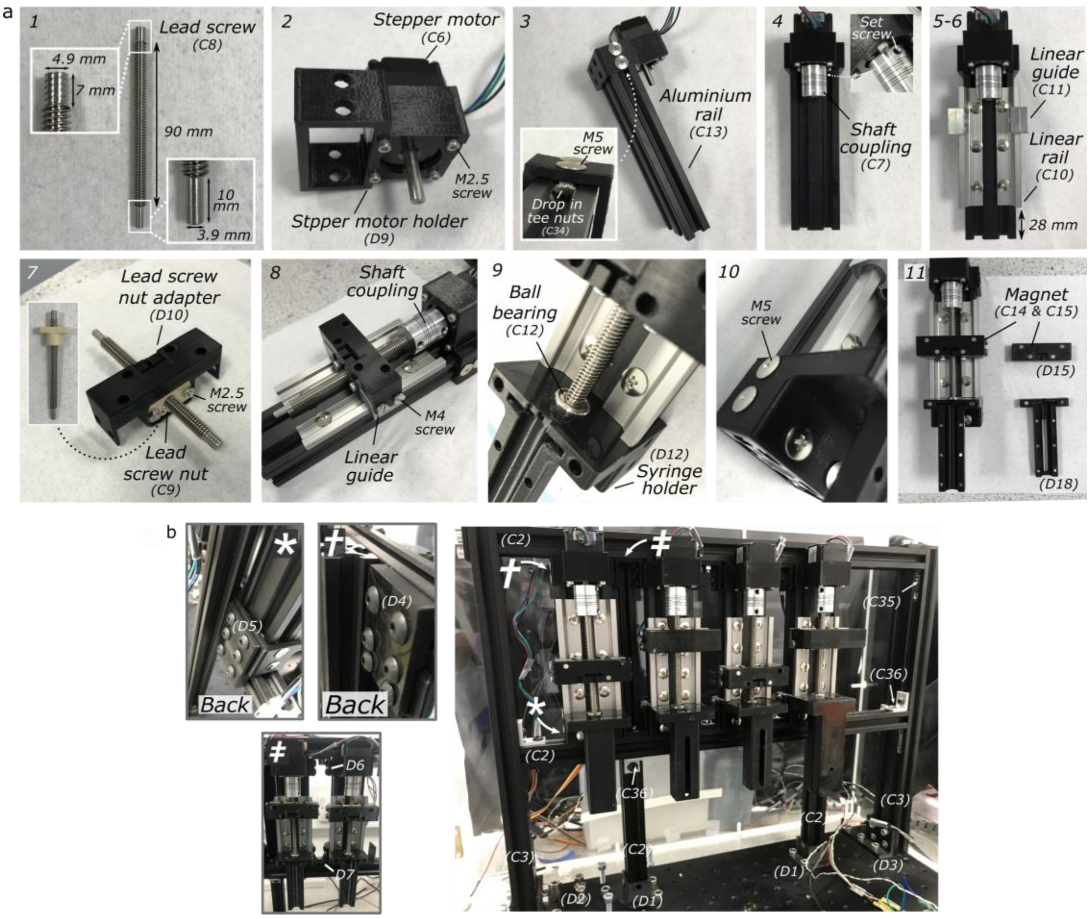

Supplementary Figure 3| Printhead assembly.

II.3 Installation of 3D printed stage to the robotic arm

All 3D printed stages were designed to mount to the uArm with an adapter piece obtained from the laser engraving kit of the uArm. To connect the stage to the uArm, first, the adapter piece was disassembled from the laser engraving kit (see **Supplementary Figure 4a**, Step 1). The adapter piece was then attached to the 3D printed stage using M3 screws (Step 2), inserted and fastened to the uArm using the hand screw of the uArm (Step 3). The uArm was locked firmly on an aluminium breadboard *(Part C4)* using clamps *(Part C5)* (Step 4).

For the heating stage, an aluminium holder *(Part D28)* was made for fitting a 3.5 mm petri dish. The aluminium holder was then wrapped with a heating coil *(Part C30)* with a K-type thermocouple *(Part C24)* placed on the holder using a high temperature tape *(Part C31)*. The holder was then clamped by two 3D printed parts *(Parts D26 & D27)* to form the heating stage (**Supplementary Figure 4b**). The installation of the heating stage to the uArm was the same as the procedure mentioned above.

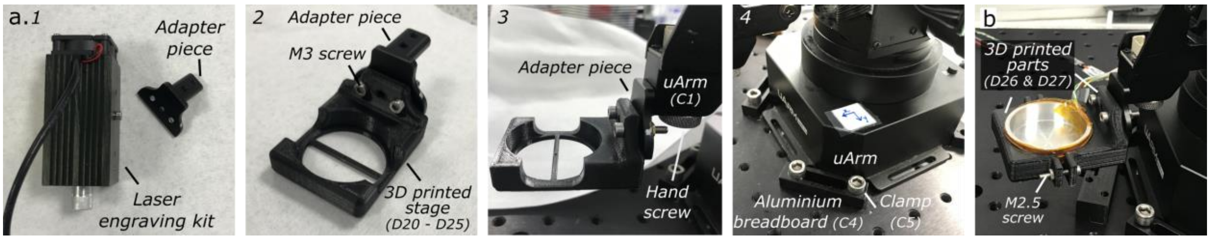

Supplementary Figure 4 | a) Assembly procedure of the stage to the uArm. **b)** A detailed view of the heating stage.

II.4 Electrical circuit of the printheads

Supplementary Figure 5 shows the circuit diagram of a printhead. The stepper motor of the printhead is controlled by an Arduino board. Before connecting the stepper motor to the Arduino board, a current limiting procedure was carried out with the motor drive to limit the loaded current. This procedure was to prevent the loaded current from exceeding the rated current of the stepper motor, hence avoiding overheating of the stepper motor. The procedure was carried as follows[39]. First, the current limit of the motor drive, I_{max}, and the reference voltage of the motor drive, V_{ref}, were calculated using the below equations. The actual current limit of the stepper motor, I_{rating}, is usually 70% of the driver current limit, I_{max}. I_{rating} is 670 mA according to the stepper motor specification[40] and R_{cs} is the current sense resistance of the motor drive, which is 0.068 **Ω** [39]**.**

$$\mathrm{I_{max}} = \frac{\mathrm{I_{rating}}}{0.7} = \frac{0.67}{0.7} = 0.96\ \mathrm{A} \tag{1}$$

$$\mathrm{V_{ref}} = 8\ \mathrm{x}\ \mathrm{I_{max}}\ \mathrm{x}\ \mathrm{R_{cs}} = 8\ \mathrm{x}\ 0.96\ \mathrm{x}\ 0.068 = 390\ \mathrm{mV} \tag{2}$$

Next, an Arduino script was written for running the motor drive in full step mode by setting the logic levels of the MS1, MS2 and MS3 pins to ‘LOW’. After ensuring the motor was not connected to the motor drive, the script was uploaded to the Arduino board. A multimeter was then used to measure the voltage, V_{ref}, between the potentiometer and the GND pin connecting to the Arduino board (see **Supplementary Figure 5**). V_{ref} was tuned to approximately 390 mV by rotating the potentiometer using a screwdriver.

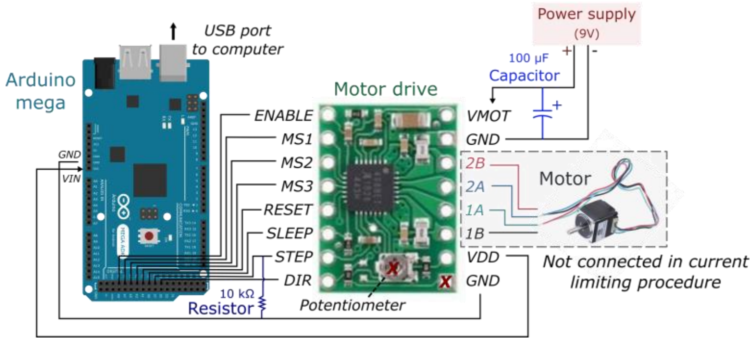

Supplementary Figure 5| Circuit diagram for connecting a stepper motor with Arduino and a motor drive. In the current limiting procedure, the stepper motor was detached from the circuit. $V_{ref,}$ was measured between the potentiometer and the GND pin to the Arduino board, as indicated by the red crosses.

After the current limiting procedure, the stepper motor was connected to the motor drive using the arrangement shown in **Supplementary Figure 5**. Overall, a single Arduino mega board and a power supply were used to control and operate four printheads. **Supplementary Figure 6** shows the overall circuit diagram. As an Arduino mega board has 54 digital pins and the control of each printhead requires 8 digital pins, more printheads can be incorporated into the system if needed.

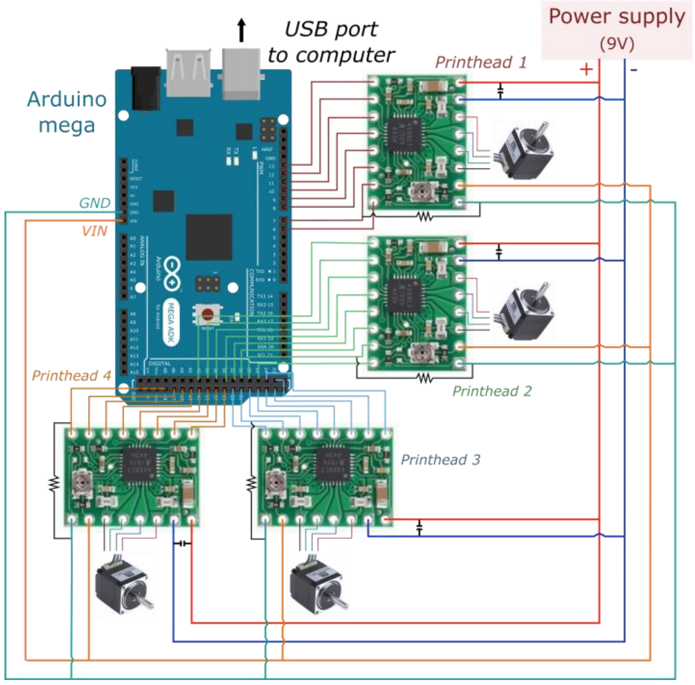

Supplementary Figure 6| Overall circuit diagram of the dispensing module composed of four printheads.

II.5 Electrical circuit of the heaters

Supplementary Figure 7 shows the circuit diagram of the heating module. The syringe heater was connected to a power adapter with rating of 9 V and 2 A, whereas the stage heater was powered by a power adapter with a 12 V and 500 mA rating. An Arduino nano board and a MOSFET were employed to control each heating module because of their compactness in size.

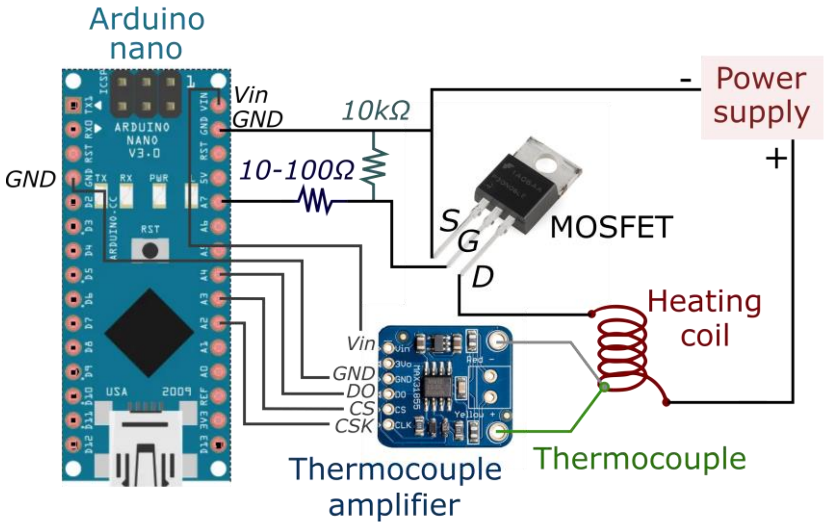

Supplementary Figure 7| Circuit diagram of a heating module.

II.6 UV module and camera

A UV light source can be easily mounted onto the aluminium breadboard when needed. Here, a low power UV light torch (5 W, *NSUV365, Nightsearcher*) with wavelength of 365 nm. A camera unit *(C922, Logitech)* was added onto the breadboard for *in-situ* monitoring and recording the printing process.

IV. Maximum build volume

The maximum build volume is estimated by subtracting the weight of the customised stage from the payload capacity of the robotic arm (see **Supplementary Table 5** which provides the estimated maximum build volume when different built stages are in use). The estimated maximum build volume is ~ 490 cm^3 (assuming the density of the material is equal to 1g/cm^3, i.e. the approximate density of most soft materials).

Supplementary Table 5| Estimated maximum build volume when different built stages are in use.

Stages	Weight of the customised stages (g)	Estimated max. build volume (cm^3)
35 mm petri dish stage	8	492
55 mm petri dish stage	15	485
90 mm petri dish stage	15	485
30 mm rectangular container stage	7	493
38 mm rectangular container stage	11	489
75x25 mm glass slide holder	10	490
35 mm petri dish heating stage	20.9	479

V. Theoretical resolution of the printhead

The theoretical volumetric resolution per step of the printhead when using a sixteenth micro-stepping resolution was calculated using below equations[23]. A microstepping stepper motor drive *(A4988, Pololu)* was chosen as it enables five microstep resolutions (full, 1/2, 1/4, 1/8 and 1/16 step)[39]. The selected stepper motor and the threaded rod have a step angle of 1.8° and a pitch spacing of 2.54 mm[40,41].

Step per revolution = 360/step angle = 360/1.8 = 200 steps per revolution

$$\textbf{Steps per mm} = \frac{1}{\text{pitch spacing}} \text{x} \frac{1}{\text{micro−stepping resolution}} \text{x steps per revolution}$$

$$= \frac{1 \text{ revolution}}{2.54 \text{ mm}} \text{ x} \frac{1}{\frac{1}{16}} \text{x} \frac{200 \text{ steps}}{1 \text{ Revolution}} = 1260 \text{ steps/mm}$$

Dispensing resolution = 1 mm/1260 steps = 0.8 µm per step

VI. Printing demonstration

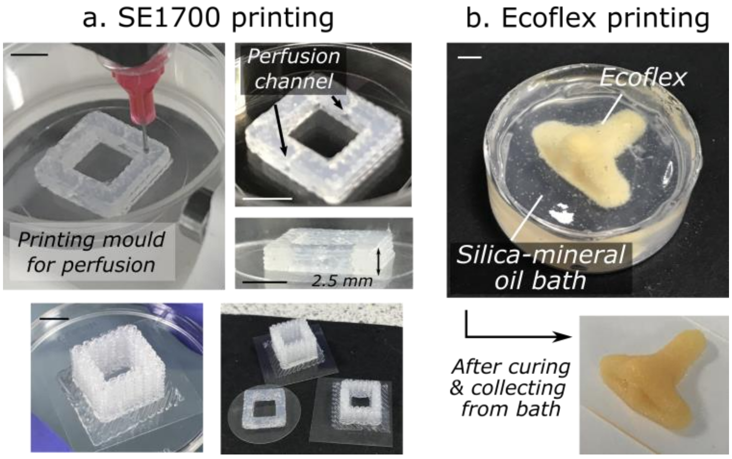

Supplementary Figure 8| Fabrication of a) perfusion moulds made of SE1700 and b) a 3D nose model made of Ecoflex using Printer.HM. Both constructs were printed using CAD files as the geometry inputs. The SE1700 ink was printed with a 25G needle, using a stage speed of 2 mm/s and an extrusion flowrate of 825 µL/h. The SE1700 construct was then cured at 70°C overnight. For the ecoflex printing, the ecoflex ink was prepared following a similar formulation reported in a previous study, where Part A ecoflex 00-30 was mixed with Part B Ecoflex 00-30 (with 1.2w/v% Slo-jo and 1.2 w/v% Thivex) at 1:1 wt. ratio with the addition of a drop of oil-based light orange color ink for visualisation. The ink was then embedded printed in a 6 w/v% fumed silica – mineral oil support bath. The printing was performed with a 27G needle, extrusion speed of 825 µL/h and a stage speed of 2 mm/s. The printing time was around 30 min. After printing, the printed construct was left at room temperature for 2 days for curing before collecting it from the bath. Scale bars = 5 mm.

VII. Theoretical linewidth calculation

The cross-sectional area, *A,* of the printed filament, when it is printed at over-extrusion condition (see **Supplementary Figure 9**), can be calculated using the following equation:

$$A = \frac{Q}{v_{dragging}}$$

where Q = Extrusion flowrate and $v_{dragging}$ = dragging speed of the filament, which can be assumed as the speed of the stage, v_{stage}. Therefore,

$$A = \frac{\pi d^2}{4} = \frac{Q}{v_{stage}}.$$

And, the theoretical line width, *d*, can be calculated as

$$d = \sqrt{\frac{4Q}{\pi \cdot v_{stage}}}.$$

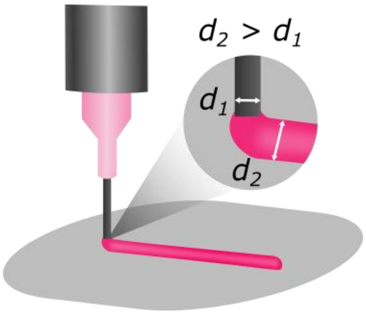

Supplementary Figure 9| Schematic showing the over-extrusion condition, where the diameter of the filament, d_2 is greater than the nozzle diameter, d_1 (die swell ratio > 1).

VIII. Codes for the printing operation

Below shows the user-defined inputs in the Python programme templates for different geometry inputs. The codes are available on Github. Users only need to define the inputs in the programme and the operation process will be executed automatically.

```
# User-defined inputs
x_line = [-10, -10, -10]  # Enter x-coordinate in mm
y_line = [-10, 0, 10]  # Enter y-coordinate in mm
spp = 30 # Printing speed
Z0 = 67.5 # Starting z position
delaytime = 30000 # Delaytime in µs between steps of the stepper motor
printhead = 3 # Assigned number of the printhead in use, 1 - 4 (4 printheads)
stage = 1 # Assigned number of the stage
          # Petri dish '1': 35 mm, '2': 55 mm, '3': 90 mm, '4': 35 mm heating stage
          # Rectangular container '5': 33 mm, '6': 40 mm
          # '7': Standard glass slide
offset = [0, 0, Z0] # Setting as [0,0,Z0] means printing at the central point (a_gcode,b_gcde) of the stage at Z0
```

Supplementary Figure 10| The required inputs in the Python template for using coordinates as the geometry input

```
# User-defined inputs
# Input equations (i.e. equation of circle)
R = 4.5
Theta = np.linspace(0, 2 * np.pi, 20)[:-1]
x_cir = R * np.cos(Theta)
y_cir = R * np.sin(Theta)
# Printing parameters
s = 0.3 # Layer height
Z0 = 66.8 # Starting z position
spp = 70 # printing speed
L1 = 1 # Thickness of the construct, mm
delaytime = 30000 # Delaytime in µs between steps of the stepper motor
printhead = 3 # Assigned number of the printhead in use, 1 - 4 (4 printheads)
stage = 1 # Assigned number of the stage
          # Petri dish '1': 35 mm, '2': 55 mm, '3': 90 mm, '4': 35 mm heating stage
          # Rectangular container '5': 33 mm, '6': 40 mm
          # '7': Standard glass slide
offset = [0, 0, Z0] # Setting as [0,0,Z0] means printing at the central point (a_gcode,b_gcde) of the stage at Z0
```

Supplementary Figure 11| The required inputs in the Python template for using equations as the geometry input.

```
# User-defined inputs
spp = 30 # Printing speed
spnp = 2000  # Speed at non-printing paths
Z0 = 67.5 # Starting z position
delaytime = 31000 # Delaytime in µs between steps of the stepper motor
printhead = 3 # Assigned number of the printhead in use, 1 - 4 (4 printheads)
stage = 1 # Petri dish '1': 35 mm, '2': 55 mm, '3': 90 mm, '4': 35 mm heating stage
          # Rectangular container '5': 33 mm, '6': 40 mm
          # '7': Standard glass slide
offset = [0, 0, Z0] # Setting as [0,0,Z0] means printing at the central point (a_gcode,b_gcde) of the stage at Z0
gcode_file = r"C:\Users\Biointerface\Documents\uArmPython\examples\Iek\Letter_P_15_15_1mm-80R-0_5s.txt" # import gcode file
```

Supplementary Figure 12| The required inputs in the Python template for using G-code generated from CAD as the geometry input. The conversion of CAD to G-code was made using 3D Slic3R, an open-source 3D slicing software.

```python
# User-defined parameters
spp =  10 # Printing speed
spnp = 2000  # Speed at non-printing paths
Z0 = 67.5 # Starting z position
delaytime = 31000 # Delaytime in µs between steps of the stepper motor
printhead = 3 # Assigned number of the printhead in use, 1 - 4 (4 printheads)
stage = 1 # Petri dish '1': 35 mm, '2': 55 mm, '3': 90 mm, '4': 35 mm heating stage
          # Rectangular container '5': 33 mm, '6': 40 mm
          # '7': Standard glass slide
offset = [0, 0, Z0] # Setting as [0,0,Z0] means printing at the central point (a_gcode,b_gcde) of the stage at Z0
# Import G-code file of picture generated from Inkscape
inkscape_file = r"C:\Users\Biointerface\Documents\uArmPython\examples\Iek\Butterfly2_0001.txt"
```

Supplementary Figure 13| The required inputs in the Python template for using picture-generated G-code as the geometry input. The conversion of picture to G-code was made using Inkscape, an open-source graphics software.

IX. Conversion of pictures to G-code

Printing with picture input was enabled by the ‘Gcodetools’ extension on Inkscape (https://inkscape.org/), which was an extension designed for CNC machines. The extension was installed on Inkscape. To covert a picture into printing paths, the workplace size on Inkscape was first adjusted according to the size of the stage by clicking ‘File → Document properties → Custom sizes’. The acceptable form of pictures can be photos of hand-drawn sketches, pictures created by any drawing software or drawings created on Inkscape. For photos of hand-drawn sketches or pictures created by other drawing software, the pictures were imported to Inkscape and were converted to a vector path using the following procedures: 1) Convert the image to a binary image (‘Filters → Color → Greyscale’) and 2) Trace the centreline of the image (‘Extensions → Images → Centerline Trace 0.8a → Select ‘Replace image with vector graphics’ and ‘Trace bright lines’). For drawings created on Inkscape, the drawing was converted to paths by clicking ‘Path → Object to path’. After converting the picture to path, the path was then placed at the centre of the workplace and was converted to a G-code file by applying the following step: 1) Navigate to ‘Extensions → Gcodetools → Orientation points’ and select ‘2-points mode’, 2) click ‘Extensions → Gcodetools → Tool library’ and select ‘Cone’; and 3) Select the path, navigate to ‘Extensions → Gcodetools → Path to Gcode’ and save the file by clicking ‘Apply’ under the ‘Path to Gcode’ tab with the file name defined in the ‘Preferences’ tab. The generated file was then imported to the Python programme for the picture input (See Supplementary Figure 12), which was designed for reading the G-code files generated by this extension.

X. Ink preparation

Supplementary Table 6| Formulations of the inks and support baths used in this work. Concentration is expressed as w/v% unless specified.

Inks	Support baths / matrices	Heating / UV	Figures
30% Pluronic F127	-	Stage heating at 40ºC	2a (Hand)
40% Pluronic F127	1.3% xanthan gum: PEGDA 700: I2959 (10:1:1 vol. ratio)	Syringe heating at 50ºC	2a (Vascular network)
68 wt% methacrylate hydroxypropyl cellulose[42]	-	UV	2a (Letter H)
PEGDA 700: DIW: I2959 (2:8:1 vol. ratio)	1% Carbopol	-	2b
Collagen	4.5% gelatin slurry	-	2b
SE1700 (base:catalyst at 10:1 wt. ratio)	-	-	2b, Supplementary Figure 8
10% Sodium carboxymethyl cellulose	-	-	2b
40% Pluronic F127	-	-	2c, 2d
SE 1700	-	-	3a (Rectangular container)
40% Pluronic F127	-	-	3a (Triangle)
40% Pluronic F127	10% gelatin + 1.3% xanthan gum	Stage heating at 40ºC	3a (Channel)
SE 1700	-	-	3b (Sine wave)
30% Pluronic F127	-	Stage heating at 40ºC	3b (Tube)
40% Pluronic F127	-	-	3b (Butterfly curve)
40% Pluronic F127	2% agarose	-	3b (Circle)
30% Pluronic F127	-	Stage heating at 40ºC	3c (Hand)
15% hydroxyapatite + 5% alginate, pre-crosslinked with 200 mM $CaCl_2$	-	-	3c (Femur)
10% Sodium carboxymethyl cellulose	-	-	3c (leaf)
10% alginate, pre-crosslinked with 200 mM $CaCl_2$	1.3% xanthan gum	-	3c (grid and Y-shaped tube)
40% Pluronic F127	1.3% xanthan gum: PEGDA 700: I2959 (10:1:1 vol. ratio)	Syringe heating at 50ºC	3d.i
40% Pluronic F127	-	-	3d.ii
25% polyacrylic acid	Fumed silica-mineral oil bath	-	3d.iii
3T3 cell suspension	-	-	4a
40% Pluronic F127	1% Carbopol	-	4b
40% Pluronic F127	-	-	4c
40% Pluronic F127	-	-	6b
3% Sodium hyaluronate	1% Carbopol	-	6c.i
10% alginate, pre-crosslinked with 200 mM $CaCl_2$	1.3% Xanthan gum	-	6c.ii – 6c.iii
Part A:Part B Ecoflex 00-30 (with 1.2w/v% Slo-jo and 1.2 w/v% Thivex) (1:1 wt. ratio)	-	-	Supplementary Figure 8

XI. Supplementary videos

Supplementary Video 1: Video showing the fabrication of a rectangular container using coordinates as the geometry input.

Supplementary Video 2: Video showing the printing of a butterfly pattern using equations as the geometry input.

Supplementary Video 3: Video showing the printing of a vascular-like structure using a CAD file as geometry input.

Supplementary Video 4: The morphing of a flower-like construct made of poly(acrylic acid) in 1M Tris solution. The flower-like construct was designed with heterogeneous printing and was printed using the geometry input of picture.

Supplementary Video 5: Dispensing of cell suspension using Printer.HM

Supplementary Video 6: Video demonstrating the non-planar printing capability of Printer.HM. A pluronic line pattern was printed on the non-planar surface of a nose model.

Supplementary Video 7: Video demonstrating the pick-and-place operation.

Supplementary References

1. Hinton TJ, Jallerat Q, Palchesko RN, et al. Three-dimensional printing of complex biological structures by freeform reversible embedding of suspended hydrogels. *Science Advances*. 2015;1(9):e1500758-e1500758. doi:10.1126/sciadv.1500758
2. Reid JA, Mollica PA, Johnson GD, Ogle RC, Bruno RD, Sachs PC. Accessible bioprinting: Adaptation of a low-cost 3D-printer for precise cell placement and stem cell differentiation. *Biofabrication*. Published online 2016. doi:10.1088/1758-5090/8/2/025017
3. Dela Nava AS, Liberos A, Nieva EG, et al. Dual extruder 3D-bioprinter for computer designed cardiac structures. In: *Computing in Cardiology*. ; 2017. doi:10.22489/CinC.2017.146-273

4. Polley C, Mau R, Lieberwirth C, Stenzel J, Vollmar B, Seitz H. Bioprinting of three dimensional tumor models: A preliminary study using a low cost 3D printer. *Current Directions in Biomedical Engineering*. Published online 2017. doi:10.1515/cdbme-2017-0028
5. Bessler N, Ogiermann D, Buchholz MB, et al. Nydus One Syringe Extruder (NOSE): A Prusa i3 3D printer conversion for bioprinting applications utilizing the FRESH-method. *HardwareX*. Published online 2019. doi:10.1016/j.ohx.2019.e00069
6. Kahl M, Gertig M, Hoyer P, Friedrich O, Gilbert DF. Ultra-low-cost 3D bioprinting: Modification and application of an off-the-shelf desktop 3D-printer for biofabrication. *Frontiers in Bioengineering and Biotechnology*. Published online 2019. doi:10.3389/fbioe.2019.00184
7. Ioannidis K, Danalatos RI, Champeris Tsaniras S, et al. A Custom Ultra-Low-Cost 3D Bioprinter Supports Cell Growth and Differentiation. *Frontiers in Bioengineering and Biotechnology*. Published online 2020. doi:10.3389/fbioe.2020.580889
8. Krige A, Haluška J, Rova U, Christakopoulos P. Design and implementation of a low cost bio-printer modification, allowing for switching between plastic and gel extrusion. *HardwareX*. Published online 2021. doi:10.1016/j.ohx.2021.e00186
9. Tashman JW, Shiwarski DJ, Feinberg AW. A high performance open-source syringe extruder optimized for extrusion and retraction during FRESH 3D bioprinting. *HardwareX*. Published online 2021. doi:10.1016/j.ohx.2020.e00170
10. Engberg A, Stelzl C, Eriksson O, O'Callaghan P, Kreuger J. An open source extrusion bioprinter based on the E3D motion system and tool changer to enable FRESH and multimaterial bioprinting. *Scientific Reports*. Published online 2021. doi:10.1038/s41598-021-00931-1
11. Koch F, Thaden O, Tröndle K, Zengerle R, Zimmermann S, Koltay P. Open-source hybrid 3D-bioprinter for simultaneous printing of thermoplastics and hydrogels. *HardwareX*. Published online 2021. doi:10.1016/j.ohx.2021.e00230
12. Cadiou T, Demoly F, Gomes S. A hybrid additive manufacturing platform based on fused filament fabrication and direct ink writing techniques for multi-material 3D printing. *International Journal of Advanced Manufacturing Technology*. Published online 2021. doi:10.1007/s00170-021-06891-0
13. Leech DJ, Lightfoot S, Huson D, Stratakos A. Low-Cost, Modular Modification to a Desktop 3D Printer for General Purpose Gel/Paste Extrusion & Direct Ink

Writing. *bioRxiv*. Published online March 11, 2021:2021.03.10.434735. doi:10.1101/2021.03.10.434735

14. Sanz-Garcia A, Sodupe-Ortega E, Pernía-Espinoza A, Shimizu T, Escobedo-Lucea C. A versatile open-source printhead for low-cost 3d microextrusion-based bioprinting. *Polymers (Basel)*. Published online 2020. doi:10.3390/polym12102346
15. Mirdamadi E, Tashman JW, Shiwarski DJ, Palchesko RN, Feinberg AW. FRESH 3D Bioprinting a Full-Size Model of the Human Heart. *ACS Biomaterials Science and Engineering*. Published online 2020. doi:10.1021/acsbiomaterials.0c01133
16. Pusch K, Hinton TJ, Feinberg AW. Large volume syringe pump extruder for desktop 3D printers. *HardwareX*. Published online 2018. doi:10.1016/j.ohx.2018.02.001
17. Lee A, Hudson AR, Shiwarski DJ, et al. 3D bioprinting of collagen to rebuild components of the human heart. *Science (1979)*. Published online 2019. doi:10.1126/science.aav9051
18. Ravi P, Shiakolas PS, Oberg JC, Faizee S, Batra AK. On the development of a modular 3d bioprinter for research in biomedical device fabrication. In: *ASME International Mechanical Engineering Congress and Exposition, Proceedings (IMECE)*. ; 2015. doi:10.1115/IMECE2015-51555
19. Xing J, Luo X, Bermudez J, Moldthan M, Li B. 3D bioprinting of scaffold structure using micro-extrusion technology. In: *Solid Freeform Fabrication 2017: Proceedings of the 28th Annual International Solid Freeform Fabrication Symposium - An Additive Manufacturing Conference, SFF 2017*. ; 2020.
20. Fitzsimmons RE, Aquilino MS, Quigley J, Chebotarev O, Tarlan F, Simmons CA. Generating vascular channels within hydrogel constructs using an economical open-source 3D bioprinter and thermoreversible gels. *Bioprinting*. Published online 2018. doi:10.1016/j.bprint.2018.02.001
21. Yenilmez B, Temirel M, Knowlton S, Lepowsky E, Tasoglu S. Development and characterization of a low-cost 3D bioprinter. *Bioprinting*. Published online 2019. doi:10.1016/j.bprint.2019.e00044
22. Lanaro M, Luu A, Lightbody-Gee A, et al. Systematic design of an advanced open-source 3D bioprinter for extrusion and electrohydrodynamic-based processes. *International Journal of Advanced Manufacturing Technology*. Published online 2021. doi:10.1007/s00170-021-06634-1

23. Shen EM, McCloskey KE. Affordable, high-resolution bioprinting with embedded concentration gradients. *Bioprinting*. Published online 2021. doi:10.1016/j.bprint.2020.e00113
24. Peng X, Kuang X, Roach DJ, et al. Integrating digital light processing with direct ink writing for hybrid 3D printing of functional structures and devices. *Additive Manufacturing*. 2021;40. doi:10.1016/j.addma.2021.101911
25. Uzel SGM, Weeks RD, Eriksson M, Kokkinis D, Lewis JA. Multimaterial Multinozzle Adaptive 3D Printing of Soft Materials. *Advanced Materials Technologies*. Published online 2022. doi:10.1002/admt.202101710
26. Shim JH, Lee JS, Kim JY, Cho DW. Bioprinting of a mechanically enhanced three-dimensional dual cell-laden construct for osteochondral tissue engineering using a multi-head tissue/organ building system. *Journal of Micromechanics and Microengineering*. Published online 2012. doi:10.1088/0960-1317/22/8/085014
27. Lee J, Kim KE, Bang S, Noh I, Lee C. A desktop multi-material 3D bio-printing system with open-source hardware and software. *International Journal of Precision Engineering and Manufacturing*. Published online 2017. doi:10.1007/s12541-017-0072-x
28. Byrne O, Coulter F, Glynn M, et al. Additive manufacture of composite soft pneumatic actuators. *Soft Robotics*. 2018;5(6). doi:10.1089/soro.2018.0030
29. Ozbolat V, Dey M, Ayan B, Ozbolat IT. Extrusion-based printing of sacrificial Carbopol ink for fabrication of microfluidic devices. *Biofabrication*. Published online 2019. doi:10.1088/1758-5090/ab10ae
30. Spiesz EM, Yu K, Lehner BAE, Schmieden DT, Aubin-Tam ME, Meyer AS. Three-dimensional patterning of engineered biofilms with a do-it-yourself bioprinter. *Journal of Visualized Experiments*. Published online 2019. doi:10.3791/59477
31. Ghosheh M, Daloo H, Usta YH, Isler Y, Karaman O. Development of innovative custom design bioprinter. In: *2016 20th National Biomedical Engineering Meeting, BIYOMUT 2016*. ; 2017. doi:10.1109/BIYOMUT.2016.7849409
32. Fortunato GM, Rossi G, Bonatti AF, et al. Robotic platform and path planning algorithm for in situ bioprinting. *Bioprinting*. Published online 2021. doi:10.1016/j.bprint.2021.e00139
33. Allevi 3 - Allevi. Accessed December 3, 2021. https://www.allevi3d.com/allevi-3/
34. Printheads - CELLINK. Accessed December 3, 2021. https://www.cellink.com/printheads/

35. BIO X 3D Bioprinter - CELLINK. Accessed December 3, 2021. https://www.cellink.com/bioprinting/bio-x-3d-bioprinter/#1599208824211-437eb779-d3ea
36. 3D-Bioplotter® Starter Series | EnvisionTEC. Accessed December 3, 2021. https://envisiontec.com/3d-printers/3d-bioplotter/starter-series/
37. Bioprinter - The modular Solution for Tissue Engineering from GeSiM. Accessed December 3, 2021. https://gesim-bioinstruments-microfluidics.com/bioprinter/
38. Zhang YS, Haghiashtiani G, Hübscher T, et al. 3D extrusion bioprinting. *Nature Reviews Methods Primers 2021 1:1*. 2021;1(1):1-20. doi:10.1038/s43586-021-00073-8
39. Allegro MicroSystems. A4988 - DMOS Microstepping Driver with Translator and Overcurrent Protection. Accessed August 7, 2021. https://www.pololu.com/file/0J450/A4988.pdf
40. RS Components Ltd. RS Pro Pro Hybrid, Permanent Magnet Stepper Motor 1.8°. Accessed August 7, 2021. https://docs.rs-online.com/6f42/0900766b8157a72a.pdf
41. drylin® lead screw, dryspin® high helix thread, right-hand thread, 1.4301 (304) stainless steel. Accessed August 7, 2021. https://www.igus.com/product/?artNr=DST-LS-6.35X2.54-R-ES
42. Lam C, Chan C, Lei M, et al. 3D Printing of Liquid Crystalline Hydroxypropyl Cellulose-toward Tunable and Sustainable Volumetric Photonic Structures. Published online 2022. doi:10.1002/adfm.202108566
